# The role and scope of practice of midwives in humanitarian settings: a systematic review and content analysis

**DOI:** 10.1186/s12960-018-0341-5

**Published:** 2019-01-14

**Authors:** Kristen Beek, Alison McFadden, Angela Dawson

**Affiliations:** 1 The Australian Centre for Public and Population Health Research, Faculty of Health, University of Technology Sydney, Sydney, Australia; 2 Mother and Infant Research Unit, School of Nursing & Health Sciences, University of Dundee, Scotland, UK

**Keywords:** Midwives, Task-shifting, Task-sharing, Humanitarian settings, Sexual and reproductive health, Disaster management cycle, Systematic review

## Abstract

**Background:**

Midwives have an essential role to play in preparing for and providing sexual and reproductive health (SRH) services in humanitarian settings due to their unique knowledge and skills, position as frontline providers and geographic and social proximity to the communities they serve. There are considerable gaps in the international guidance that defines the scope of practice of midwives in crises, particularly for the mitigation and preparedness, and recovery phases. We undertook a systematic review to provide further clarification of this scope of practice and insights to optimise midwifery performance. The review aimed to determine what SRH services midwives are involved in delivering across the emergency management cycle in humanitarian contexts, and how they are working with other professionals to deliver health care.

**Methods:**

Four electronic databases and the websites of 33 organisations were searched between January and March 2017. Papers were eligible for inclusion if they were published in English between 2007 and 2017 and reported primary research pertaining to the role of midwives in delivering and performing any component of sexual and/or reproductive health in humanitarian settings. Content analysis was used to map the study findings to the Minimum Initial Service Package (MISP) for SRH across the three phases of the disaster management cycle and identify how midwives work with other members of the health care team.

**Results:**

Fourteen studies from ten countries were included. Twelve studies were undertaken in conflict settings, and two were conducted in the context of the aftermath of natural disasters. We found a paucity of evidence from the research literature that examines the activities and roles undertaken by midwives across the disaster management cycle. This lack of evidence was more apparent during the mitigation and preparedness, and recovery phases than the response phase of the disaster management cycle.

**Conclusion:**

Research-informed guidelines and strategies are required to better align the scope of practice of midwives with the objectives of multi-agency guidelines and agreements, as well as the activities of the MISP, to ensure that the potential of midwives can be acknowledged and optimised across the disaster management cycle.

**Electronic supplementary material:**

The online version of this article (10.1186/s12960-018-0341-5) contains supplementary material, which is available to authorized users.

## Introduction

In 2015, 65.6 million people were forcibly displaced worldwide, with data indicating that more than 125 million people were in need of humanitarian assistance globally [1]. Seventy-five percent of those in need of humanitarian assistance are women and girls, aged 15 to 49 [2]. A lack of access to sexual and reproductive health (SRH) services and information is a leading cause of morbidity and mortality among displaced women and girls of reproductive age [3]. Sixty percent of preventable maternal deaths and 45% of neonatal deaths take place in displacement settings [4] and more than one in five refugees or displaced women in humanitarian settings experience sexual violence [5] placing them at risk of unplanned pregnancies, HIV and other sexually transmitted infections (STIs).

Motivated healthcare staff able to deliver SRH services are required throughout the health emergency and disaster risk management cycle. Before a crisis, planning and preparation may include working with communities for disaster risk reduction (DRR) and building partnerships, capacity and supply systems. The Minimum Initial Service Package (MISP) for Reproductive Health in Humanitarian Settings [6] outlines five objectives, activities and resources to address the SRH needs of people in humanitarian emergencies. In the post-crisis transition and recovery phase, skilled staff are needed to restore and strengthen SRH services to reduce vulnerability to future events [3]. However, there is a global shortage of health workers in general, estimated at 7.2 million in 2013 [7], and of skilled birth attendants in particular. The World Health Report [8] reported a global shortage of 2.4 million midwives, nurses and physicians among 57 countries below the threshold for 80% coverage of skilled birth attendants. In this context, it is unsurprising that there is also a dearth of skilled health workers during humanitarian crises [9, 10]. This shortage includes both coverage [9] and lack of health workers with the necessary skills [9–11]. Midwives, trained to international standards and competencies, are associated with improved quality of care and efficient use of resources [12] and can potentially meet some of the shortfall of skilled health workers in humanitarian crises.

The geographic and social proximity of midwives to the communities they serve has the potential to prevent avoidable maternal deaths [13] and has been recognised as a key strength of this cadre. Global momentum toward increasing midwifery numbers and, in some contexts, introducing midwives as a defined sector in the health workforce are further evidence of the potential contribution of midwifery to sexual and reproductive health [14–16]. Importantly, there is significant alignment between the scope of midwifery practice and the objectives and activities of the international response to SRH in humanitarian settings [17, 18]. This indicates that midwives, working alongside other health professionals and providers, have the potential to meet the sexual and reproductive health needs of people in conflict and disaster contexts, as they do in stable settings.

One review examined community midwifery workforce issues such as recruitment and retention in fragile and conflict-affected countries, drawn largely from grey literature [19]. However, there are no reviews providing insight into midwives’ scope of practice in the field or how this relates to international guidance.

Guidance on the potential scope of midwifery practice during humanitarian emergencies can be found in position statements released by the International Confederation of Midwives (ICM) and a report from the World Health Organization (WHO) [20]. One ICM position statement [21] encourages midwives to continue providing ongoing care and support to women during childbirth and to breastfeeding women, suggesting that the scope of practice of a midwife as defined by the ICM [22] is applicable to disaster contexts.

However, when the statements provided by ICM and WHO are compared against the roles and scope of practice of the SRH health workforce in crisis as outlined in multi-agency technical guidelines and consensus documents [17, 23–25], considerable gaps exist. Table 1 provides an overview of potential workforce roles extracted from this documentation across the emergency disaster management cycle (mitigation, preparedness, response and recovery). The table identifies six areas of workforce practice before an emergency. However, little specific guidance is provided for midwives by the ICM and WHO across these areas with gaps noted in how midwives can be best engaged in population-based health education, delivering preventative measures and mobilising community members to monitor hazards. While more information is available to guide midwives in their response efforts, considerable gaps can be seen in the recovery phase of the disaster cycle. Guidance on how midwives can contribute to sustaining, consolidating and expanding SRH services, and further developing partnerships and synergies with humanitarian and development actors to ensure comprehensive SRH care and services are provided, is absent.

**Table 1:** Potential midwifery roles across the emergency disaster management cycle according to key guidelines and statements

	Before an emergency: mitigation and preparedness					
SRH in crisis multi-agency guidelines	Integrate SRH into disaster risk reduction/ mitigation, emergency preparedness and response plans Make sure SRH is included in disaster risk reduction/ mitigation, emergency preparedness and response plans: allocate human and financial resources to see this happen.	Address laws, policies and practices that affect whether people in crises can access SRH services: a. Address laws, policies and practices for SRH for stable settings: Build resilience by addressing SRH in laws, policies and practices for stable settings. (WHO [3]; UNISDR [25]) b. Assess laws, policies and practices for SRH in crises: Review and develop national and local frameworks of laws, regulations and policies, ensuring the coherent integration of SRH and DRR. (UNISDR [25]) c. Include vulnerable populations: Develop and adopt specific policies and practices for the inclusion of women, adolescents, newborn, displaced, disabled and other vulnerable groups in humanitarian settings. (WHO [3]). d. Clarify and implement coordination policies and procedures: Ensure clear policies are in place at all levels for the coordination of SRH services and supplies (WHO [3]).	Involve the community, particularly vulnerable groups in monitoring: Develop and implement early warning systems by establishing community networks to monitor hazards, vulnerabilities and capacities at a local level. (WHO [3])	Identify and reduce risks for vulnerable communities and SRH services by reducing underlying risk factors…“by ensuring strong primary health care and preventive health measure with key provisions for SRH (and advance gender equality)” (WHO [3]:3).	Identify and prepare human resources, infrastructure, funding, & supply, information and logistics systems. a. Identify and estimate capacity: Locate and assess existing human resources for SRH (WHO [24]) b. Engage existing resources: “Prepare existing SRH services to absorb impact, adapt, respond to and recover from emergencies” (WHO [3]:3) by integrating disaster risk management into primary, secondary and tertiary health care (UNISDR [25]:4/6). c. Develop relevant curricula/ training courses: Systematically include health emergency risk management, emergency response planning and the MISP in the curricula for SRH workers, and health and emergency management workers more broadly. (WHO [3]:3). Enhance training capacities in disaster medicine and encourage the implementation of disaster risk reduction approaches in health work. (UNISDR [25]) d. Ready supplies: Pre-position SRH supplies, including reproductive health kits in support of MISP implementation	“Undertake population-based health education around the needs of women and babies before, during and after birth with a particular emphasis on danger signs and when and where to seek care” (WHO [24]:2). “Integrated SRH messages into health sector and non-health sector-driven public awareness campaigns and educational materials about disaster risk management” (WHO [24]:2).
Midwifery scope of practice WHO/ ICM	Midwives included in strategic disaster planning and midwifery integrated into disaster risk reduction/ mitigation, emergency preparedness and response plans: a. Advocacy: ICM urges Member Associations to advocate to institutions and government for the inclusion in disaster preparedness and response plans for midwifery services and the equity and social justice elements needed to deliver these services. (ICM [21]) b. Strategic Planning: ICM and WHO encourage the active participation of midwives in strategic disaster preparedness and response planning activities with institutions and government. (ICM [21]: Position statement 2014_003). (WHO [20]: 8).	Address laws, policies and practices that affect whether people in crises can access midwifery services: a. Assess and develop laws, policies and practices: ICM and its Member Associations will work in leadership and partnership with involved organisations to address legal, policy and practice support for access to midwifery services in crises. (ICM [21]); (ICM [65]: b. Ensure equity and access: ICM will amplify the voice of women and children affected by disasters by advocating to ensure equity and equality in access to health services during and directly after a disaster. (ICM [21]: Position statement 2014_003)			Identify and prepare midwives to be effective in disaster/ emergency situations a. Provide information and facilitate training: ICM will “[p]romote the dissemination and facilitate access to knowledge, information, and training on disaster/emergency preparedness for midwives” (ICM [21]: Position statement 2014_003 p2). b. Understand local disaster typologies: ICM encourages Member Associations to familiarise themselves with local disaster/ emergency realities and associated health needs, and to disseminate this understanding to members. c. Systematically train midwives to be effective in emergency situations: Midwives should be prepared to plan for and respond to disasters by incorporating disaster/ emergency preparedness and response into current curricula, and providing continuing education opportunities on disaster midwifery. (ICM [21]: Position statement 2014_003)	
	During an emergency: response					
The MISP	Ensure an organisation is identified to lead the implementation of the MISP; -RH Officer in place -Meetings to discuss RH implementation held -RH Officer reports back to health cluster/ sector -RH kits and supplies available and used	Prevent and manage the consequences of sexual violence; -Protection system in place especially for women and girls -Medical services and psychosocial support available for survivors -Community aware of services	Reduce HIV transmission; -Safe and rational blood transfusion in place -Standards precautions practiced -Free condoms available	Prevent excess maternal and newborn death and illness; -EmONC services available − 24/7 referral system established -Clean delivery kits provided to birth attendants and visibly pregnant women -Community aware of services	Plan for comprehensive sexual and reproductive health care, integrated into primary health care, as the situation permits. -Background data collected -Sites identified for future delivery of comprehensive RH -Staff capacity assessed and trainings planned -RH equipment and supplies ordered	Additional priorities: a. Continue family planning b. Manage symptoms of STIs c. Continue HIV care and treatment d. Distribute hygiene kits and menstrual protection materials
Disaster Midwifery Scope of Practice: ICM &WHO	Leadership: “Regardless of command structure it is often the person on the scene who takes initial leadership” (WHO [20]:8).	Sexual Violence: “…assist in efforts to mobilise the necessary resources for midwifery care in disaster/emergency situations, giving special attention to vulnerable groups” (ICM [21]: Position Statement 2014_003 p3). WHO list of core competencies for nurses and midwives in emergencies includes: “Practical competencies to treat people with special needs, i.e. vulnerable groups and addressing gender-based violence” (WHO [20]:10).	Reducing HIV transmission a. Standard precautions in care during pregnancy, labour and post-partum period: “…the principles of infection control often need to be emphasised” (WHO [20]: 6) “ICM…believes that Personal Protective Equipment (PPE)- latex gloves etc.- should be available to midwives at an affordable cost” (ICM [66]). “Midwives are urged to accept their responsibility [by]…Following universal precautions when handling body fluids and at other times of handling infected or potentially infected blood or blood stained products” (ICM [66]: Position Statement PS2008_006). b. Minimising transmission of HIV during birth: “Working in partnership with medical staff and women agreeing the optimum method of birth to minimise mother-to-foetus transmission” (ICM [66]: Position Statement PS2008_006). c. PMTCT: “Midwives are in a unique position to support breastfeeding and safe infant feeding during times of natural disaster or emergency, thereby protecting the health of infants in these circumstances” (ICM [21]: Position Statement 2014_003 p3). “Working in partnership with women to determine the optimum method of feeding the newborn to prevent vertical transmission, and providing support for the implementation of the woman’s choice of feeding method” (ICM [66]: Position Statement PS2008_006). d. Free condoms for post-partum health and general sexual and reproductive health: “ICM…urges midwives, in their capacity as professionals and members of communities to be educators as well as practitioners in working to prevent the spread of HIV and provide care and treatment as it becomes available” (ICM [66]: Position Statement PS2008_006).	Prevent excess maternal and neonatal mortality and morbidity: “ICM urges Member Associations with regard to disaster/ emergency *response* to:” -Encourage midwives to continue to provide ongoing care and support to women during childbirth [which encompasses pregnancy, birth and the postnatal period], and to lactating women. -Work with existing capacities, skills, resources, and organisational structures. -“Care for midwives and others who provide direct services” (ICM [21]: Position Statement 2014_003 p3). “A key gap is responding to the psychosocial needs of nurses and midwives affected by emergencies…” (WHO [20]: 15).	Planning and collecting background data: “ICM will: …-Contribute to assessments and reports on MNCH during and after disasters/emergencies through partnerships with other relevant organisations and international networks” (ICM [21]: Position Statement 2014_003 p2). WHO list of core competencies for nursing and midwifery in emergencies includes: “competencies for needs assessment and planning, providing and managing care: situation and needs assessment” (WHO [20]: 10).	Additional priorities: a. Midwives involved in family planning: “ICM supports the right of women to control their pregnancies, and takes every opportunity at regulatory, educational, and political level to enhance this right by…” -participating in the strategic planning, provision and evaluation of services which enable women to plan their pregnancies and prevent unwanted; -ensuring all women have available to them family planning services which are appropriate, accessible, cost-effective (or free of charge), and women-friendly; -providing quality advice and support to women in a way and at a level which is relevant to their needs; -“…strengthening midwives role in pre-conceptual health education for adolescent and school age groups to prevent unplanned and adolescent pregnancies” (ICM [67]. b. Clinical care during pregnancy, labour and post-partum include managing symptoms of STIs and/or ARVs (ICM [66], 2008: Position Statement PS2008_006: “ICM…underlines that all HIV positive pregnant women have a right to access anti-retroviral drugs for themselves and their newborns”).
	After an emergency: protracted crises and recovery					
SRH in Crisis Granada Consensus	Mainstream SRH in all health policies: Integrate and mainstream SRH in all health policies and strategies to revitalise and strengthen the health system during recovery.	Achieve sustainable consolidation and expansion of SRH: Build upon the minimum standards provided by the MISP in a contextually appropriate way. Consider human resources, capacity development, local and district level operations and the coverage of SRH services as they support consolidation and expansion.		Develop partnerships and synergy between humanitarian and development actors: Prevent gaps and loss of SRH services as the crisis moves from acute to post-acute phases through partnerships between development and humanitarian actors. This should include ensuring funds and a commitment to sound health recovery plans, policies and strategies.	4. Recognise and support local leadership: Develop policies and strategies that recognise and support the leadership role of national and local authorities, communities and beneficiaries in ensuring SRH.	
Disaster Midwifery Scope of Practice: ICM &WHO	Advocacy and strategic planning				Midwifery leadership	

While there are gaps in the guidelines to clarify the roles and scope of practice of midwives to deliver SRH in crisis settings that may constrain the contribution of midwifery, less is known about the practice of midwives in the field. Furthermore, there is ambiguity in the delineation of roles and the relationship between SRH cadres such as midwives, doctors, community health workers and lay health workers [26]. This ambiguity is partly underpinned by the inconsistent use of the term “midwife” to refer not only to fully trained and regulated midwives as defined by the ICM but in many settings is used for lesser skilled workers such as auxiliary midwives or unsupported, poorly trained midwives [12, 27]. In many settings, the scope of practice of midwives is limited by the dominance of the medical profession [28–30]. In many Asian and South American countries, midwives are non-existent or have been marginalised, leading to a lack of understanding among other health care professionals and policymakers, of their scope of practice [12, 27]. Homer et al. [31] highlighted the unexploited potential of collaborative practice to improve SRH.

This lack of knowledge is problematic for the effective engagement of midwives within the broader humanitarian health sphere and for developing the disaster competency of the midwifery workforce. To begin to address this lack of evidence, we conducted a systematic review to examine the roles and relationships of midwives in humanitarian settings to inform a comprehensive approach to maximising the potential of midwives in such contexts. We sought to identify and describe the SRH care activities, services and resources midwives are involved in delivering in humanitarian emergency contexts, and how they work with other care providers to deliver health care.

## Methods

### Study design

The systematic review was conducted using an a priori protocol. An initial scoping exercise identified relevant databases and websites where literature on SRH in humanitarian settings, the roles of midwives in humanitarian settings, human resources for humanitarian health, disaster planning and response, and the SRH of displaced populations could be retrieved. This broad initial approach also assisted in the identification of keywords for our more focused search. As a result of the scoping exercise, four electronic databases and the websites of 33 organisations were systematically searched. These electronic bibliographic databases and websites are listed in Table 2, as are the keywords employed in our search. Reference lists of key documents were hand-searched for additional resources.

**Table 2:** Sources and keywords

Sources	Keywords
Electronic bibliographic databases	Midwifery OR (health care) manpower OR nurse midwives OR maternal health services OR delivery, obstetrics (obstetric delivery) OR maternal mortality OR midwifery workforce. AND Emergencies OR emergency responders OR emergency medical (health) services OR emergency medical technicians OR disaster planning or civil defence OR emergency medicine OR disasters OR disaster victims OR disaster medicine OR disaster planning OR crisis intervention OR relief work OR refugees OR humanitarian.
MEDLINE, Embase, Scopus and Science Direct	
NGO websites	
American Refugee Committee, CARE, International Consortium for Emergency Contraception, International Medical Corps, International Planned Parenthood Federation- The SPRINT Initiative, Ipas, The International Rescue Committee, Jhpiego, John Snow, Inc., Population Action International, Save the Children, Women’s Refugee Commission, Cambridge Reproductive Health Consultants, CHANGE: Centre for Health and Gender Equity, Cordaid, Gynuity Health Projects, Medicins du Monde, Inter-agency Working Group on Reproductive Health in Crises, RAISE, Marie Stopes International, International Federation of Red Cross and Red Crescent Societies, International Confederation of Midwives and Direct Relief	
Research organisations	
Columbia University - The Heilbrunn Department of Population and Family Health, Centre for Reproductive Rights, Emory University, Human Rights Centre- University of California Berkeley School of Law, University of Technology Sydney, George Washington University- Global Women’s Institute, Guttmacher Institute, The Centres for Disease Control and Prevention	
United nations agencies	
United Nations Children’s Fund, United Nations High Commissioner for Refugees, United Nations Population Fund, World Health Organization	

### Study selection and appraisal

SRH care was defined as including those activities, services and resources outlined in the MISP [17]. We considered research pertaining to humanitarian emergency contexts, described as circumstances in which any hazard, including armed conflict, threats of natural origin, political repression, epidemics, technological hazards or a complex combination of these [32], results in a crisis of any type or scale. Key documentation was used to define areas of midwifery work in the mitigation/preparedness phase of a crisis [3, 17, 24], SRH activities in the response phase [17] and areas of action in recovery [23].

We defined the scope of practice of midwives in line with the definition provided by the ICM [22] and The State of the World’s Midwifery report [18] that confirm the midwife’s involvement with broader SRH.

A diverse range of research evidence was sought for this study and qualitative, quantitative and mixed methodological research included. The inclusion and exclusion criteria are presented in Table 3.

**Table 3:** Inclusion and exclusion criteria

Included	Excluded
In English	In languages other than English
Contemporary papers (years 2007–2017)	Pre 2007
Papers reporting primary research (of any method)	Papers reporting other forms of research including literature reviews
Papers pertaining specifically to the work of midwives	Papers pertaining to clinical staff whose primary function is not to provide midwifery services
Papers which differentiate the work of midwives from other cadre	Papers which discuss the roles of “skilled birth attendants” or “SRH staff” without differentiation by cadre
Papers pertaining to the role of midwives in delivering and performing any component of sexual and/or reproductive health outlined in the MISP, clinical and/or non-clinical	Papers pertaining to general/other components of health care
Papers including a description of the role of midwives in delivering SRH care in humanitarian emergency contexts and/or how they work with other health professionals to deliver SRH care in humanitarian emergency settings	Papers in which the role(s) of midwives are not described, or where involvement of midwives/ midwifery skills is recommended not implemented. Papers pertaining to the role of midwives in meeting the SRH needs of refugee women in country of resettlement
Papers addressing any point in the continuum of an emergency (mitigation, preparedness, response and recovery)	Development settings and where the humanitarian setting is not directly described or addressed within the paper

**Fig. 1:**
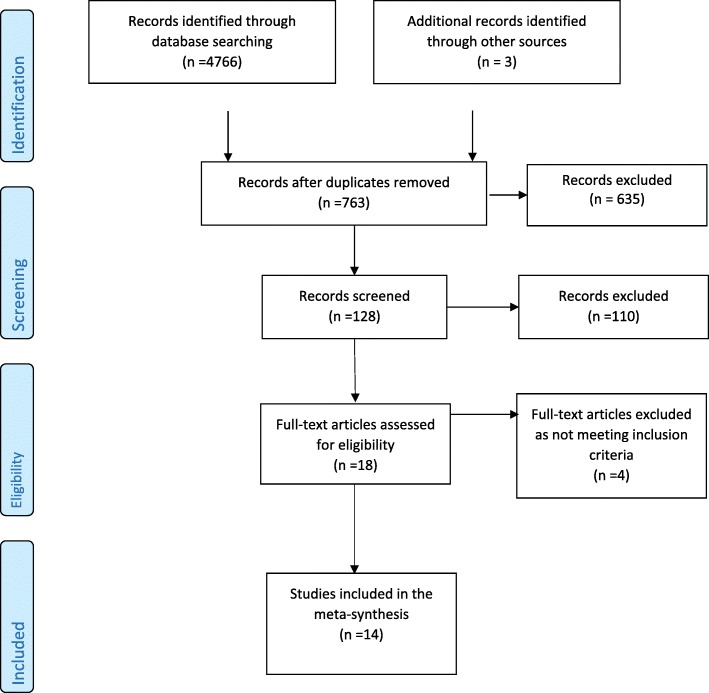
Study identification and selection

Searches were conducted between January and March 2017 and results managed using Endnote software. Two researchers (KB and AD) screened titles and abstracts; full texts were obtained from relevant papers, and these were screened by two reviewers (KB and AD) against the inclusion/exclusion criteria. Differences were resolved through discussion with a third reviewer (AM). The systematic review process adhered to the Preferred Reporting Items for Systematic Reviews and Meta-Analysis (PRISMA) [33] guidelines (see Fig. 1).

Eighteen papers meeting the inclusion criteria were critically appraised by three researchers using the Critical Appraisal Skill’s Programme assessment tools for qualitative research [34] and Pluye et al.’s [35] scoring system for appraising mixed methods research, and the National Institute for Health and Care Excellence (NICE) guidelines [36] for survey and cross-sectional studies. Four studies were excluded from the review [37–40] as they did not adequately state research aims or methodology used.

### Data extraction and synthesis

All data in the findings sections of the included 14 papers were extracted for analysis. We applied content analysis to categorise data in a systematic and replicable way [41]. The phases of the disaster or emergency management cycle represented in Table 1 provided a framework to organise emergent findings on the roles and relationships of midwives in natural, conflict and protracted crisis settings. We further explored the involvement of midwives in the delivery of SRH activities according to multi-agency guidance, WHO/ICM scope of practice, the MISP, Granada Consensus and cross cadre collaboration. Tables were created to identify and explore patterns across the findings sections of the papers under review. These were discussed by the authors to reach consensus.

## Findings

Fourteen papers met our selection criteria and were included in this review (see Additional file 1 for details of included studies). Ten studies employed qualitative methods [42–50] and two were each based on quantitative [51, 52] or mixed methodologies [53, 54]. As detailed in Table 4, eight papers reported findings from the response phase of the disaster, including one instance of immediate response within protracted humanitarian settings [42]. Findings were reported on the recovery phase in seven papers, and of these, one paper pertained to both the response and recovery stages of intervention [49]. Two studies closed the cycle between humanitarian and development work by detailing the roles of midwives in both preparedness and recovery [53, 54].

**Table 4:** Characteristics of documents included in review

Study	Disaster phase			Disaster type			Reported SRH involvement of midwives										
	DRR/preparedness	Response	Recovery	Natural	Conflict	Protracted crisis	Routine MNH	BEmOC	B/CEmNC	CEmOC	PMTCT	FP	SV	EC	PAC/SAC	ASRH	STSTI
Bosmans et al. [42]		✓			✓	✓	✓					✓					
Chi et al. [43]			✓		✓		✓	✓	✓		✓						
Chi et al.		✓			✓		✓									✓	
Furuta and Mori [44]			✓		✓	✓	✓					✓					
Hobstetter et al. [45]		✓			✓		✓						✓	✓			
Lee [46]		✓			✓		✓					✓					
McGready et al. [51]		✓			✓		✓										
O’Malley Floyd [53]	✓		✓	✓			✓										
Oyerinde et al. [52]			✓		✓		✓	✓		✓							
Speakman et al. [47]			✓		✓		✓	✓	✓			✓	✓				
Sugino et al. [48]		✓		✓			✓					✓					
Tappis et al. [49]		✓	✓		✓		✓	✓							✓	✓	
Turkmani et al. [54]	✓		✓		✓	✓	✓					✓			✓		
Wick and Hassan [50]		✓			✓		✓	✓									

Acronyms:

*DRR* disaster risk reduction, *MNH* maternal, newborn health, *BEmOC* basic emergency obstetric care, *B/CEmOC* basic and comprehensive emergency obstetric care, *CEmOC* comprehensive emergency obstetric care [68], *ANC* antenatal care, *IPC* intrapartum care, *PNC* postnatal care, *FP* family planning, *SV* sexual violence, *EC* emergency contraception, *PAC/SA* post-abortion care/safe abortion, ASRH adolescent sexual and reproductive health, *PMTCT* prevention of mother-to-child transmission of HIV, *STSTIs* syndromic treatment of sexually transmitted infections

Twelve papers were from settings of conflict and two described the aftermath of natural disasters. According to the WHO regions, five studies were from the Eastern Mediterranean region, four each were from Africa and Southeast Asia, and one was from the Americas. Finally, and accounting for the multiple foci of some studies, 13 papers described the roles of midwives in providing maternal and newborn health services, three detailed this cadre’s role in providing family planning, one described general reproductive health work and one discussed interventions to address sexual violence and provide emergency contraception. The characteristics of the included studies are summarised in Table 4 and discussed below according to both the three phases of the emergency management cycle and the guidance and areas of action associated with each according to multi-agency guidelines, the MISP and the Granada Consensus (see Table 5).

**Table 5:** Midwifery roles and practice identified across the emergency disaster management cycle according to key guidelines and statements

	Before an emergency: mitigation and preparedness					
SRH in crisis multi-agency guidelines	Integrate SRH into disaster risk reduction/mitigation, emergency preparedness and response plans	Address laws, policies and practices that affect whether people in crises can access SRH services:	Involve the community, particularly vulnerable groups in monitoring:	Identify and reduce risks for vulnerable communities and SRH services by reducing underlying risk factors	Identify and prepare human resources, infrastructure, funding, and supply, information and logistics systems.	Undertake population-based health education
Review findings	X	X	X	X	Pre and in-service training of midwives	X
	During an emergency: response					
The MISP	Ensure an organisation is identified to lead the implementation of the MISP	Prevent and manage the consequences of sexual violence	Reduce HIV transmission	Prevent excess maternal and newborn death and illness	Plan for comprehensive sexual and reproductive health care, integrated into primary health care, as the situation permits	Additional priorities: a. Continue family planning, b. Manage symptoms of STIs, c. Continue HIV care and treatment, d. Distribute hygiene kits and menstrual protection materials
Review findings	X	Provision of ECP by midwives	Infection control and PMCTC	ANC, BEmOC, BEmNC, CEmOC, PNC ASRH, Referral, linking communities and health services	X	ART Family planning
	After an emergency: protracted crises and recovery					
SRH in Crisis Granada Consensus	Mainstream SRH in all health policies:		Achieve sustainable consolidation and expansion of SRH:	Develop partnerships and synergy between humanitarian and development actors:	Recognise and support local leadership:	
Review findings	X		Training and recruitment of midwives	X	X	

Key: X = No evidence from review

### Before an emergency: mitigation and preparedness

Evidence was only found in one area of work as per the multi-agency guidelines, i.e. preparedness training of midwives (see Table 5). Two studies reported training of midwives in different humanitarian settings [53, 54].

In Afghanistan, Turkmani et al. [39] evaluated a pre-service competency-based midwifery curriculum comprising clinical components for antenatal, labour and postnatal care, and family planning services. Programme graduates indicated teaching was conducted in a culturally sensitive way, observed local customs and provided material support to trainee-midwives from remote areas. O’Malley Floyd [53] reported expatriate midwives, primarily from the United States of America, training local midwifery staff before and after the 2010 Haiti earthquake. The training provided by the non-profit organisation “Midwives for Haiti” was based on WHO’s midwifery education modules, American College of Nurse-Midwives Lifesaving Skills manuals and two Hesperian Foundation publications [53] and aimed to address high maternal and infant mortality.

### During an emergency: the Minimum Initial Service Package

We found evidence in eight papers of midwifery staff being involved in delivering routine maternal newborn care, basic emergency obstetric care, family planning and adolescent SRH services. These activities represent action in only four of the six objectives of the MISP (Table 5).

#### MISP objective 2: Prevent sexual violence and assist survivors

The only reference to activity related to preventing or responding to sexual violence in humanitarian settings concerned the low level of knowledge about the use of emergency contraception for both survivors of sexual violence and broader populations among midwives, on the Thailand-Burma border [45].

#### MISP objective 4: Prevent excess maternal and neonatal mortality and morbidity

Six of the seven papers reporting on the response phase of an emergency [43, 48–51] and a paper which detailed the response and recovery phases [49] discussed the role of midwives in providing maternal and newborn health services. Midwives were reported as being involved in births at both health facilities and in women’s homes [42, 46, 48, 50, 51]. Birthing at home was often supported by midwifery staff when access to facilities was compromised by ongoing conflict [42, 49, 50]. Examples of the challenges faced by midwives in conflict situations included in Gaza assisting a mother to birth in a house that was shaking from heavy shelling [50] and being required to stay and attend to women at a health facility during periods of curfew [42]. Tappis et al. [49] reported examples from Afghanistan of midwives lacking skills, e.g. administration of magnesium sulphate for pre-eclampsia and manual vacuum extraction, and lack of equipment, and both constraints on referral for women experiencing obstetric emergencies, and over-referral. No further clarification of the skills midwives applied to their roles in terms of the specifics of emergency obstetric and neonatal care signal functions was provided.

Although outside the scope of the MISP, examples of midwives working within multi-disciplinary teams in the Philippines and Thailand to provide antenatal care (ANC) during periods of conflict were described in two papers [46, 51]. Chi et al. reported that the incursion of violence in their study site had meant that “ANC attendance was largely a luxury for many women” and that a lack of attendance for antenatal care was associated with an increase in pregnancy and birth complications.

Also beyond the scope of the minimum response outlined by the MISP, midwives were reported as being involved in postnatal care in some settings, e.g. the Philippines [46], and for adolescent women during conflict in Buruindi and Uganda. Bosman et al. [42] reported that comprehensive postnatal care was compromised in Palestine due to the ongoing conflict as curfews and checkpoints restricted staff access.

#### Additional priorities

Of the additional priorities of the MISP, only family planning was discussed as being part of midwives’ work in the response phase of emergency contexts. Lee [46] reported that two NGOs working in conflict-affected Maguindanao made modern contraceptives and family planning services available, although the specific role of midwives among a wider team of SRH service providers was unclear. Bosmans et al. [42] explained the difficulty of providing family planning services in the longstanding humanitarian crisis in Palestine as such programmes were perceived as population control programmes.

### After an emergency: recovery and protracted settings

Of the four strands of action relating to the recovery phase outlined in the Granada Consensus, evidence of only one (midwives roles in the expansion and consolidation of SRH) was identified in the findings of seven papers that reported on activities in protracted emergencies and recovery after a crisis (see Table 5).

The role of midwives in both home and facility-based birth was discussed. In Burundi and Uganda, for example, while midwives and medical doctors comprise the key emergency obstetric and neonatal care providers, severe capacity gaps were noted in the provision of newborn emergency care [43]. Both Chi et al. [43] and Furuta and Mori [44] reported that midwives are trusted to attend births and to recognise the signs of obstetric emergencies and, as such, are in high demand. For women seeking care in a refugee camp in Sudan, there is a cultural preference for homebirths and midwives are not always able to leave the facility to attend [44]. In such instances, traditional birth attendants may be approached and health care from village midwives is only sought for serious conditions.

Oyerinde et al. [52] described signal functions that state-registered midwives performed in post-conflict Sierra Leone: administering parenteral antibiotics, oxytocics and anticonvulsants, manual removal of placenta, removal of retained products, assisted vaginal delivery and blood transfusion. However, the availability of these functions and the capacity of midwifery staff to provide them were not uniform across the study area. Chi et al. [43] reported that in post-conflict Northern Uganda, staff shortages impact on midwives’ involvement in maternal and newborn health activities including antenatal care, births, the Early Infant Diagnosis room, the anti-retroviral treatment clinic, *prevention of mother-to-child transmission* (PMTCT) of HIV and emergency care.

Three studies discussed the training of midwives in the post-crisis recovery phase. O’Malley Floyd [53] and Turkmani et al. [54] report training of midwives before the crisis or in the aftermath of an emergency with the purpose of strengthening the health system. Speakman et al. [47] discuss the consistently positive contribution the Community Midwifery Education programme in Afghanistan has had on the reduction of maternal mortality and the increase in skilled attendance at birth.

### Midwifery collaboration with other health cadre

Other health workers cited as fellow SRH service providers across the emergency management cycle include doctors, traditional birth attendants, nurses, anaesthetists, laboratory technicians, surgeons, medical assistants, maternal and child health aides, health volunteers, medical technologists, sonographers, and computer programmers.

Four papers reported that traditional birth attendants are an established source of SRH information and services, as being involved in births either at home [42, 44], at facilities [52] or at places unspecified. In all cases, however, inadequate numbers of skilled birth attendants had resulted in either a reliance on or re-emergence of traditional birth attendants. As explained by Bosmans et al. [42], a cut to external funding in Palestine had meant that “there was no other option than to reintegrate the dayats [traditional birth attendants] for home births and postnatal visits, even though some of them had not received any training”.

The relationship between nurses and midwives was discussed in-depth in only one study. Speakman et al. [47] report that midwives and nurses worked collaboratively in the aftermath of a natural disaster in Indonesia. Midwives were required to undertake more generalist nursing duties such as the care of trauma cases in addition to their regular duties, and both nurses and midwives were tasked with increased administrative responsibilities [48]. Importantly, midwives called for clarification of the scope of practice for nurses and midwives [47]. The need for midwives to provide services not related to maternal and newborn health in emergency contexts was also reported by Turkmani et al. [54].

Speakman et al. [47] and Turkmani et al. [54] characterise the relationship between midwives and doctors by a lack of recognition and an infringement on midwives’ self-perceived scope of practice. Midwives reported that doctors in Afghanistan often failed to acknowledge the skills of qualified midwives and discouraged them from performing some tasks [47]. Afghan midwives also reported feeling discriminated against by other providers, especially doctors, and were frustrated by restrictions placed upon their scope of practice [54]. Conversely, midwives in Gaza felt a sense of solidarity in their work with physicians [50].

Chi et al. [43] reported a lack of coordination between key emergency obstetric and neonatal care personnel in post-conflict Northern Uganda, particularly when needing to assemble a team of skilled providers to perform an emergency caesarean section, causing delays in important lifesaving services. At a broader level, midwives in Central Java in the aftermath of a natural disaster [48] raised concerns about coordination between midwives and nurses, and the many agencies providing disaster relief to their communities. Midwives and nurses expressed that they were not adequately informed of the activities of relief medical operations in the area [48].

## Discussion

The aim of this review was to synthesise evidence regarding the roles midwives play in humanitarian contexts and their relationship with other cadres involved in the provision of SRH information and services in these settings. Across the 14 studies, we found examples of activities undertaken by midwives during the three phases of the emergency management cycle. However, as indicated in Table 5, our review clearly demonstrates that there are important gaps between the levels of guidance provided to midwives and their organisations relating to mitigation and preparedness, and recovery. Furthermore, there were substantive gaps between guidance and evidence of on-the-ground practice in all three phases of the disaster management cycle.

There is some alignment between international guidance on what is required in humanitarian settings and what the ICM/WHO asserts is the scope of midwifery practice, particularly during the response phase despite gaps in the research evidence of midwifery activity in two areas of the MISP. We did not find guidance from ICM or WHO on the potential role midwives may play in the mitigation and preparedness phase in relation to involving the community, particularly vulnerable communities, in monitoring or in reducing risks for vulnerable communities by reducing underlying risk factors, or in providing population-based health education. Evidence from two papers that reported data on preparing midwives for disasters shows that little is known about the preparation of midwives to deliver the MISP but also in terms of how midwives are involved in integrating SRH into disaster risk reduction/mitigation preparedness and response planning and in to laws that ensure equity and access. We did not find any guidance from ICM or WHO on the potential role midwives may play in the recovery phase in relation to achieving sustainable consolidation and expansion of SRH, or in developing partnerships and synergy between humanitarian and development actors. The research evidence provides even less information on actual practice in the field. Given that the WHO recommends that nurses and midwives are supported to operate with a greater scope of practice in emergencies [20], this lack of guidance for important aspects of the emergency cycle is significant. Without such guidance, midwives are unlikely to deliver care to their full potential across all phases of the disaster management cycle.

In terms of collaboration and conflict with co-workers, there were three main issues: the re-emergence of traditional midwives due to shortage of skilled birth attendants, collaboration of midwives and nurses resulted in midwives assuming care that is beyond the scope of midwifery practice, and tensions between midwives and medical personnel resulting in limits to the scope of midwifery practice. Such tensions were reported in Afghanistan, possibly reflecting a lack of recognition of the role of midwives who are re-emerging as a profession in Afghanistan, as well as gender inequality [55, 56]. We did not find any research that focussed directly on describing the role of midwives in relation to other cadres. Such research could inform future guidance for midwives operating in these contexts and seeking to contribute to building the resilience of their communities.

Our review found evidence of midwife shortages that led to the re-emergence of traditional birth attendants, lack of support for midwives and challenges around lack of recognition of the role and scope of midwives’ practice. In many countries, these issues are not specific to emergency settings [12, 27] but reflect wider issues. There is an urgent need to scale-up all midwifery training to address shortages and to ensure that midwives have the necessary knowledge and skills to function to their full potential in non-emergency contexts as well as across all phases of emergency management. In contexts where there is a lack of midwives, a short-term solution could be to train other healthcare workers such as nurses, doctors and community health workers to provide aspects of SRH [57]. However, considerable investment is needed globally to prioritise midwifery and build capacity so that midwives are a highly competent, qualified workforce as per ICM standards [18, 27, 58].

Lessons from successful midwifery training in emergencies must be shared and scaled up, especially those reported directly from the field [55, 59]. However, while there are excellent clinical packages available to assist the training of midwives to deliver the MISP including the SRH clinical outreach refresher training (S-CORT) [60] and m-health applications [61], supportive strategies such as supervision are necessary to enable midwives to transfer their learning into practice [62]. Midwifery education for humanitarian training must also be incorporated into basic education and training. Leadership is necessary to achieve this. While the voices of midwives need to be represented at high-level international fora, the ICM is well positioned to support professional associations of *midwives in nations across* the world to strengthen quality midwifery education, care guidelines and protocols for emergencies. Midwifery associations and regulatory bodies and the Ministries of Health particularly those in LMIC can be supported by country offices of United Nations agencies such as WHO and UNFPA to better locate the roles of midwives in high-level disaster preparedness and response planning and coordination activities. The Inter-Agency Working Group for Reproductive Health (IAWG) is also an important player to assist coordination with humanitarian actors and provide technical guidance.

Implementation research is required to address the evidence gap concerning the effective delivery of midwifery-led sexual and reproductive health care in crises settings by testing workforce interventions to support midwives to deliver the Minimum Initial Service Package and strengthen collaboration and referral pathways. Workforce interventions need to be assessed alongside interventions to strengthen the health information system to generate data to inform immediate and future responses to SRH in crisis [63]. Finally, these need to be adequately costed so that financing benchmarks can be established to mobilise, accumulate and allocate money to cover the SRH needs [64].

A limitation of this review is language bias as we only included papers written in English. The search returned few papers highlighting the emerging nature of this research field and as such decisions were made to include studies that despite being methodologically “weak” provided important contextual data. The review did not allow for theorising due to the largely descriptive nature of the data. However, the use of a framework for analysis derived from the phases of emergencies and international guidelines has delivered useful insights for midwifery policy and practice in humanitarian settings.

## Conclusion

This systematic review identified considerable gaps in the guidance that defines midwifery scope of practice in crises in ICM and WHO documentation particularly for the mitigation and preparedness, and recovery phases of an emergency. As shown in Table 5, there is a lack of evidence that examines midwifery interventions across the disaster management cycle, in particular, during mitigation and preparedness, and recovery phases. Research-informed guidelines and strategies are required to better align the midwifery scope of practice with the objectives of multi-agency guidelines and agreements, as well as the activities of the MISP to ensure that the potential of midwives can be acknowledged and optimised across the disaster management cycle.

## Additional file

Additional file 1: **Table S6.** Summary of studies included in the review. (DOCX 44 kb)
