# Supplementary material for: The role and scope of practice of midwives in humanitarian settings: a systematic review and content analysis
Source: Hum Resour Health. 2019 Jan 14;17:5. doi: 10.1186/s12960-018-0341-5 (PMC6333021; doi:10.1186/s12960-018-0341-5)
Supplement: Supplementary file 1 — Table S6. Summary of studies included in the review. (DOCX 44 kb) [file 12960_2018_341_MOESM1_ESM.docx]

**Additional File 1**

Table S6 Summary of studies included in the review

| Reference | Country | Methods | Aim of paper | Role of midwives | Summary of Findings |
| --- | --- | --- | --- | --- | --- |
| ([Bosmans et al. 2008](#_ENREF_1)) | Palestine | Qualitative:In-depth interviews with key informants from 19 organisations and two focus group discussions were carried out in the West Bank and Gaza. Three refugee camps visited as well as five health facilities. | “The aim of this paper is to highlight that the complexity of the Israeli–  Palestinian conflict is seriously affecting the sexual and reproductive rights of both refugee and  non-refugee women in the West Bank and Gaza” (p103). | Midwives and doctors working in very remote areas to provide home health services.  Policies on attendance at delivery that had aimed to replace TBAs with trained midwives had had to be reconsidered. “There was no other option than to reintegrate dayats [TBAs] for home deliveries and post-natal visits, even though some of them had not received any midwifery training” (p107).  In one health centre the interview was organised with the nurse and the midwife, the only people remaining from the former staff, forcibly reduced due to conflict.  One public health centre was no longer attended by a physician due to repeated curfews and checkpoints so services were provided by the local midwife and an assisting nurse who could only do some general follow-up of the mothers and their babies (measuring and weighing).  Health staff at the maternity unit of a referral hospital were required to stay at the hospital during periods of curfew so that they could attend to women already there.  NGO established a hotline (staffed by volunteers) which would identify a doctor or midwife to attend home deliveries.  Training of midwives on home deliveries and providing them with clean delivery kits. | The findings revealed that severe restrictions on mobility had reduced access to health facilities for both staff and patients in a significant way. Lack of donor interest and withdrawal of donor support mentioned as hampering implementation. Family planning had become a politically sensitive issue, and there were indications of increased gender-based violence. |
| ([Chi et al. 2015a](#_ENREF_2)) | Burundi & Uganda | Qualitative: Qualitative comparative case study that used 42 face-to-face semi-structured in-depth interviews and 4 focus group discussions for data collection. Participants were 32 local health providers and 37 staff of NGOs working in the area of maternal health. Data was analysed using the framework approach. | “The aim of this study was to explore the barriers to effective delivery of EmONC services in post-conflict Burundi  and Northern Uganda, in order to provide policy makers and other relevant stakeholders context-relevant data on improving the delivery of these lifesaving services” (p1/24). | Midwives reported as being one of the 2 major cadres of EmONC providers (along with medical doctors).  Doctors and midwives have been trained in managing emergency obstetric care.  Many health-centres III and upwards “have at least one midwife who is trained in early newborn care and management of the postnatal period” (p8/24).  High demand on midwives (due to lack of staff, high turnover etc.): “In facilities with only one midwife, she may have to work all year round without a period of leave” (p12/24).  Midwives “handling antenatal clinic, deliveries, the EID (Early Infant Diagnosis) room, the ART clinic, PMTCT and emergency” (p12/24)- mentioned in context of human resource challenges and overwork of midwifery staff.  Midwives feel unrecognised for their work compared to other cadres. Also feel unsupported and demotivated (p12/24).  Poor coordination amongst key EmONC personnel- particularly in assembling teams of doctors, nurses, midwives etc. to perform an emergency caesarean (p13/24).  The critical role midwives play in these settings is discussed (p19/24). | The availability, quality and distribution of EmONC services were major challenges across the sites. The barriers in the delivery of quality EmONC services were categorised into two major themes; human resources-related challenges, and systemic and institutional failures. |
| ([Chi et al. 2015b](#_ENREF_3)) | Burundi & Uganda | Qualitative:  Descriptive qualitative study that used in-depth interviews (IDIs) and focus group discussions (FGDs) with women, health providers and staff of NGOs for data collection. | The aim of this study is to explore the effects of armed conflict on maternal and reproductive health in Burundi and Northern Uganda. | Does not differentiate between cadres of health workers, with this exception:  -Midwives involved in provision of ASRH: “midwives reported that teenage girls between 12-18 years from disadvantaged backgrounds are particularly vulnerable to unintended pregnancies and constituted over half of their clients” (p7/15).  Training of midwives interrupted due to insecurity (p6/15).  Mentions re-emergence of TBAs due to unavailability of SBAs including midwives (p7/15). | The main themes that emerged from the study were: armed conflict as a cause of limited access to and poor quality of MRH services; armed conflict as a cause of poor MRH outcomes; and armed conflict as a route to improved access to health care. |
| ([Furuta & Mori 2008](#_ENREF_5)) | Sudan | Qualitative:  Questionnaires, in-depth interviews and informal conversations, analysing findings with the PRECEDE-PROCEED model. | Furuta and Mori (2008) aim to provide understanding of the broad range of potential factors which affect risk behaviours related to safe motherhood among refugee women in Eastern Sudan. | Midwives work with doctors, medical assistants and nurses within a long standing refugee camp. “The village midwives were the key workers responsible for maternal and neonatal care, providing delivery care mainly at home” (886).  Midwife involvement with, and refusal to perform, FGC within the camp (p892).  Health volunteers, not midwives, are main providers of FP information. Mentions “low quality of FP services with a lack of counselling skills (health workers themselves felt ashamed discussing sexuality)…” (pp896-897).  Midwives are preferred as delivery attendants (perceived as more skilled, having clean equipment, same cost as TBA, able to refer etc.). This preference extends to pregnancy-related decision makers (generally husband and mother-in-law). But, preference is for home births and midwives are not always available for this (p897 & 899). TBAs accessed in these cases. “Only when the condition is perceived to be serious will they seek health care from village midwives, regardless of the place of birth” (p899).  Inadequate midwifery staff impedes skilled birth attendance, as do inadequate supplies/ resources.  Village midwives act as a bridge between traditional and modern medicine. “They can visit women’s homes to maintain the traditional custom as well as attend patients in the health centre, thus creating trust in midwives’ skills that can be viewed as a part of the modern medical system” (p898).  Midwives are trusted to perform deliveries and recognise the signs of obstetric emergencies.  Midwives do not have time to undertake continued studies/ refresher courses (p901). | Factors frequently were uncontrollable for an individual woman, suggesting the importance of a supportive political, social, and educational environment for safe motherhood. |
| ([Hobstetter et al. 2015](#_ENREF_6)) | Thai-Burma border | Qualitative:  Multi-methods qualitative assessment among cross-border populations, migrants, and refugees. 46 key informant interviews with representatives from 25 organizations, 18 focus group discussions with migrant adults, migrant adolescents, and healthcare workers, and a service mapping exercise with 22 stakeholders. | The objective of this study was to evaluate availability, service delivery, and barriers to access to emergency contraceptive pills  on the Thailand–Burma border. | Midwives have little knowledge/ are misinformed about the safety and use of ECPs. | Low use of ECPs among the target populations. Structural barriers and lack of evidence-based reproductive health protocols, education, and information restrict access to the limited family planning resources available in this region. Misinformation about ECPs was widespread among health workers and organizational policies were often non-evidence based. |
| ([Lee 2008](#_ENREF_10)) | Philippines | Qualitative:  This exploratory study describes the experiences of local government service providers and two NGOs in a context of long-standing internal armed conflict, how and to what extent provision has been affected by the conflict and what has been done to overcome its effects. It is based on interviews with six health service coordinators and providers (including midwives). | The aim of this study is to describe “the experiences of local government service providers and two NGOs in a context of  long-standing internal armed conflict, how and to what extent provision has been affected by the conflict and what has been done to overcome its effects” (p65). | Three tier service provision model. Provincial level- hospital; municipal level- rural health unit; village level- village health station. “One medical doctor serves 3-4 municipalities, one midwife 3-4 villages and one volunteer health worker for every village health station” (p68). Services provided by NGO 1 in conflict affected region (by a team consisting of medical doctor, nurse, midwife, medical technologist and computer programmer): modern contraceptives, Pap smears, antenatal, delivery and post-natal services, and counselling.  Providers “follow where our patients evacuate to- day and night. We always offer quality services even there…We are sensitive to evacuees’ needs…” (p69).  Services provided by NGO 2 in conflict affected region (utilising provincial health office facilities): antenatal, natal and post-natal services and FP. Also followed evacuees to new sites and provided services (does not specify if by midwives).  Both organisations provide services regardless of political affiliations. | Collaboration for maternal health care provision is effective. Service delivery structures have generally been spared from direct attack due to the perceived benefits and neutrality of services. When displacement occurs due to conflict, service providers follow the population to continue care. |
| ([McGready et al. 2012](#_ENREF_11)) | Thailand | Quantitative:  All medical records from women who attended the Shoklo Malaria Research Unit antenatal clinics from 12(th) May 1986 to 31(st) December 2010 were reviewed, and maternal death records were analyzed for causality. | This study reports  on maternal deaths within a system of weekly antenatal clinics offering frequent screening for early detection and treatment of malaria for refugees and migrant women. | Trained midwives assist delivery at the Shoklo Malaria Research Unit (SMRU). Antenatal care, HIV testing, PTMTC and malaria screening also provided (though not clear if by midwives). SMRU facilities are open 24 hours a day and are staffed by locally trained medical assistants, nurses, midwives, sonographers, laboratory technicians, home-visitors, support staff and a small number of expatriate doctors” (p3/10). | Frequent antenatal clinic screening allows early detection and treatment of falciparum malaria and substantially reduces maternal mortality from P. falciparum malaria. No significant decline has been observed in deaths from sepsis or other causes in refugee and migrant women on the Thai-Myanmar border. |
| ([O'Malley Floyd 2013](#_ENREF_13)) | Haiti | Mixed methods:  Three questionnaires were developed to survey Haitian students, staff midwives, and volunteers who served with Midwives for Haiti. Questions were designed to elicit information about how well the volunteers were prepared for their experience, the effectiveness of translation services, and suggestions for improving the preparation of volunteers and strengthening the education program. | “The purpose of the study was: 1) to develop a description of the program’s strengths and its deficits in order to determine if there was a need to improve the preparation of volunteers prior to service and 2) to make recommendations aimed at strengthening the volunteers’ contributions to the education of Haiti and auxiliary midwives” (p558). | Expatriate volunteer midwives, predominantly from the USA were recruited by the organisation to train skilled birth attendants in Haiti. These services expanded following the 2010 earthquake. Volunteer midwives were involved in attending clinics, didactic teaching and supervision of students in the hospital.  Have now developed a web-based pre-deployment training programme for volunteers. | Analysis of the surveys of volunteers, staff, midwives, and the Haitian students generated several common themes. The 3 groups agreed that the volunteers made an effective contribution to the program of education and that the volunteer midwives need more preparation prior to serving in Haiti. The 3 groups also agreed on the need for better translators and recommended more structure to the education program. |
| ([Oyerinde et al. 2011](#_ENREF_14)) | Sierra Leone | Quantitative:  From June to August 2008, a cross-sectional survey was conducted of health facilities in Sierra Leone offering delivery services. Assessment tools were local adaptations of tools developed by the Averting Maternal Death and Disability program at Columbia University, New York, USA. | The aim of this study was to conduct a needs assessment for emergency obstetric care in order to address high maternal and newborn mortality in Sierra Leone 8 years after the end of the civil war. | Registered midwives involved in providing the following signal functions: administering parenteral antibiotics, oxytocics and anticonvulsants; manual removal of placenta; removal of retained products; assisted vaginal delivery; and blood transfusion (though not uniformly across the study area- for example, only 28% of CHCs and hospitals had midwives able to perform assisted vaginal delivery).  Other health personnel involved in SRH services in CHCs and hospitals included medical officers; MCH aides/ TBAs/ community health nurses- none of whom meet the definition of SBA.  Inadequate numbers of SBAs in this setting resulted in the authors’ recommendation for training, recruitment and retention of midwives. In response, the government and its partners established a rural midwifery school and increased enrolment into existing midwifery schools. | There were enough comprehensive EmOC (CEmOC) facilities in the country but they were poorly distributed. There were no basic EmOC (BEmOC) facilities. Few facilities assisted vaginal delivery (AVD), and 3 potentially BEmOC facilities did not meet the standard only because they did not perform AVD. Severe shortages in staff, equipment, and supplies, and unsatisfactory supply of utilities severely hampered the delivery of quality EmOC services. Demand for maternity and newborn services was low, which may have been related to the poor quality and the high/unpredictable out-of-pocket cost of such services. |
| ([Speakman et al. 2014](#_ENREF_15)) | Afghanistan | Qualitative:  This case study analyses CME development and implementation to help determine successes and challenges. Data were collected through documentary review and key informant interviews. Content analysis was informed by Walt and Gilson's policy triangle framework. | “The aim of this study was to assess how the CME [Community Midwifery Education] programme developed, implemented, and accepted in Afghanistan. It explores its potential impact on maternal health and more broadly as an example of female empowerment” (p2/12). | Key skills midwives were required to develop by the MoPH:  “-the full range of midwifery care, including antenatal, delivery and postnatal care;  -management of complications according to basic emergency obstetric and newborn care principles;  -newborn and infant care;  -selected reproductive health care (e.g. contraceptives);  -linkages between women/ families/ communities/ health facilities” (p6/12).  Some midwives also involved in home based delivery.  Reports that doctors fail to acknowledge the skills of qualified midwives and discourage them from performing some tasks. | The CME programme has contributed to consistently positive indicators, including up to a 1273/100,000 reduction in maternal mortality ratios, up to a 28% increase in skilled deliveries, and a six-fold increase in qualified midwives since 2002. Begun as a small pilot, CME has gained support of international donors, the Afghan government, and civil society. |
| ([Sugino et al. 2014](#_ENREF_16)) | Indonesia | Qualitative:  Focus group discussions and questionnaire survey were conducted with 11 nurses and 11 midwives of public health centres in Bantul. Content analysis was applied to analyse transcripts of the focus group discussions and the responses to questionnaire. | “The purpose of this paper is to identify the nurses’ and midwives’ perceptions and understanding of their roles, as well as the needs of training in disaster preparedness and management” (p420). | Village midwives are primarily responsible for maternity and health needs of children, including monitoring and check-ups.  Nurse and midwives were rarely informed of the activities of relief agencies working in the disaster area.  During the response, midwives were “required to care for trauma cases in the same way as nurses in addition to their regular maternity responsibilities” (p427).  Nurses and midwives worked collaboratively- “Collectively they worked tirelessly taking care of patients suffering from the effects of the disaster” (p428).  Nurses felt, however, that midwives were more respected for their work as they were able to conduct their practice more independently.  Nurses and midwives identified the need for disaster preparedness training for themselves and disaster preparedness activities with the community.  In addition to their clinical duties, nurses and midwives were also tasked with increased administrative demands.  Both nurses and midwives agreed that they “must work together in post-disaster health service beyond their routine work assignments” (p430).  Authors call for clarification of the roles and responsibilities of nurses and midwives in disaster response. | Health care for survivors and community were provided by highly committed health professionals supported in strong community resilience. Donors driven relief programs tended to be unorganized and insensitive for local health providers. Organized disaster management trainings are strongly needed to develop disaster nursing and preparedness. |
| ([Tappis et al. 2016](#_ENREF_18)) | Afghanistan | Qualitative:  Interviews with community members; service providers; and district, provincial and national officials. Data was then triangulated with programme and policy documentation to identify factors that affect the coverage of safe delivery and emergency obstetric care services. | The aim of this paper is to explore factors that affect the availability and utilisation of intrapartum care services in four districts of Afghanistan. | Respondents report good intrapartum care provided by midwives in health clinics but stress the barriers for women in attending such facilities due to ongoing conflict and other challenges.  Unequal distribution of midwives able to administer magnesium sulphate for pre-eclampsia together with inconsistent protocol on midwives administering this drug led to referral by midwives.  In some areas, MVAs are available but midwives skilled in their use are not, while in other places the converse is true.  Midwives are more generally involved in decisions to refer for obstetric complications. | Comparison of barriers to maternal health service coverage across the four districts highlights the complexities of national health policy planning and resource allocation in Afghanistan, and provides examples of the types of challenges that must be addressed to extend the reach of life-saving maternal health interventions to women in fragile and conflict-affected states. |
| ([Turkmani et al. 2013](#_ENREF_19)) | Afghanistan | Mixed methods:  The evaluation used a mixed methods approach that included qualitative and quantitative components. This paper focuses on the qualitative components which included in-depth interviews with 138 graduated midwives and 20 key informants as well as 24 focus group discussions with women. | The objective of this study was to evaluate a pre-service midwifery education program through the identification of its strengths and weaknesses. | Midwives felt the training was strong in terms of antenatal/postnatal care, management of normal labour and delivery, management of retained placenta, breech deliveries and FP.  Midwives reported feeling less confident in terms of management of pre-eclampsia/ eclampsia, shock, and haemorrhage.  Midwives reported “being socially recognised and appreciated by other co-workers for their competency. Many midwives also reported opportunities to share their updated knowledge and clinical skills with others. Some respondents spontaneously mentioned mentoring doctors and medical students…” (p1168).  Midwives report the importance of cultural sensitivity and collaboration with the community. They have been involved in outreach activities within communities, including working with men.  Midwives reported incongruence between the standards taught and those observed in clinical settings (e.g. compliance with infection prevention practices).  “At times, communities requested midwives to provide care that was beyond their scope of practice” (p1169).  “The majority of practicing midwives reported feeling discriminated against by other providers, especially doctors…Almost all midwives practicing where doctors also worked expressed frustration at the restrictions placed upon their scope of practice…Furthermore, the midwives felt frustrated by job descriptions that limited their ability to prescribe simple medications for women’s general health problems. Team work was also noted to be a challenge” (p1169).  Midwives also reported the challenge of heavy caseloads, including provision of non- MNH/RH services. | Midwives and stakeholders perceived that women were more likely to use maternal and child health services in communities where midwives had been deployed. Strengths included evidence-based content, standardised materials, clinical training, and supportive learning environment. Weaknesses of the programme included perceived low educational requirement to enter the programme and readiness of programmes to commence education. Insecurity and geographical remoteness are perceived as challenges with clients' access to care and the ability of midwives to make home visits. |
| ([Wick & Hassan 2012](#_ENREF_31)) | Palestine | Qualitative:  This study, based on interviews with women who gave birth and midwives during the 22-day Israeli attack on Gaza in December 2008 - January 2009, illustrates the vulnerability and trauma women experience when there is no safe place for childbirth. | The aim of this study is to illustrate “the vulnerability and trauma women experience when there is no safe place for childbirth” (p7) by exploring birthing experiences of women and midwives in Gaza. | Midwives involved in delivery at home and in hospital/clinic settings, and community education on danger signs of obstetric emergencies.  Midwives felt they were under-resourced and “ill-equipped as they did not have the basic skills, materials and confidence to provide care in the community. They had no birthing kits or emergency supplies, and they did not feel protected by the system in case of complications” (p12).  Midwives reported that they worked in solidarity with physicians and other hospital workers. | Most midwives were unprepared both materially and psychologically to attend births outside a hospital setting, while physicians were overwhelmed with severely injured patients. The capacity of midwifery care to keep birth normal whenever possible is particularly crucial in situations of political instability, conflict, poverty and disaster. |
